# Structural and Intermediate Social Determinants of Health Linked to Adolescent Pregnancy in the Peruvian Rural Context

**DOI:** 10.3390/ijerph23081067

**Published:** 2026-08-18

**Authors:** Suli Sánchez Gómez, Yshoner Antonio Silva-Diaz, Ruth Clarivel Vega-Rojas, Cintya Elisabeth Odar-Rojas, José Luis Rodriguez Medina, Miuller Raul Muñoz Zumaeta, Hitler Adolfo Vela Zuta

**Affiliations:** 1 Grupo de Investigación Plantas Medicinales y Medicina Alternativa (PYMA), Centro de Investigación Plantas Medicinales, Terapias Alternativas y Comunidades Nativas y Rurales (CIPMAYCOM), Instituto de Salud Integral Intercultural (ISI), Facultad de Ciencias de la Salud (FACISA), Universidad Nacional Toribio Rodríguez de Mendoza (UNTRM), Chachapoyas 01001, Amazonas, Peru; 2 Escuela Profesional de Psicología, Facultad de Ciencias de la Salud (FACISA), Universidad Nacional Toribio Rodríguez de Mendoza (UNTRM), Chachapoyas 01001, Amazonas, Peru; 3 Facultad de Derecho y Ciencias Políticas, Universidad Nacional Toribio Rodríguez de Mendoza (UNTRM), Chachapoyas 01001, Amazonas, Peru; 4 Facultad de Ciencias Económicas y Administrativas, Facultad de Ciencias de la Salud (FACISA), Universidad Nacional Toribio Rodríguez de Mendoza (UNTRM), Chachapoyas 01001, Amazonas, Peru

**Keywords:** social determinants of health, adolescent mothers, pregnancy, pregnancy in adolescence, alcohol drinking

## Abstract

**Highlights:**

**Public health relevance—How does this work relate to a public health issue?**

- Adolescent pregnancy in Latin America is a global public health issue, with higher rates in rural areas, where the population faces barriers to accessing healthcare services and sexual education.
- Maternal and neonatal complications associated with adolescent pregnancy generate a significant mortality burden in multiple countries, impacting social and economic development and the sustainability of healthcare systems in Latin American countries with rural Amazonian settings.

**Public health significance—Why is this work of significance to public health?**

- Health insurance type, extreme poverty, and parents’ educational level are health determinants associated with adolescent pregnancy, and understanding these factors highlights the need for differentiated policies in rural Amazonian settings.
- The finding that the level of contraceptive knowledge is associated with a lower prevalence of pregnancy, while the effective use of contraceptives is not associated, reveals a gap in access to knowledge and healthcare systems in rural settings.

**Public health implications—What are the key implications or messages for practitioners, policy makers and/or researchers in public health?**

- It is of great importance to strengthen access to sexual and reproductive health education services by coordinating extreme poverty reduction policies and improving public health insurance coverage.
- The study’s findings open a largely underexplored line of research in which the mechanisms that hinder the translation of knowledge into behavioral adoption—such as perceptions of contraceptives or gender roles—are examined. Future research should address the heterogeneity of socially determined factors as conceptualized within cultural contexts.

**Abstract:**

Background: Adolescent pregnancy is a public health problem in Latin America, with a higher prevalence in rural areas. This population faces barriers to accessing education and healthcare services, increasing the risk of maternal and neonatal complications. Methods: This study employed a cross-sectional analytical design and included 72 adolescents aged 12 to 17 years in Lonya Grande. The associations between structural and intermediate social determinants of health and the prevalence of adolescent pregnancy were assessed. Results: Among the structural determinants, extreme poverty (PR: 2.48; 95% CI: 1.23–5.03; *p* = 0.004), type of public health insurance (PR: 3.05; 95% CI: 1.21–7.72; *p* = 0.005), and parental educational level were associated with a higher prevalence of adolescent pregnancy. After adjustment, having public health insurance (aPR: 0.49; 95% CI: 0.36–0.66; *p* < 0.001) and having parents who had completed primary school (aPR: 1.67; 95% CI: 1.15–2.42; *p* = 0.01) were associated with a higher prevalence of adolescent pregnancy. Among the intermediate determinants, alcohol consumption (PR: 2.77; 95% CI: 1.85–4.16; *p* < 0.001), knowledge about contraceptives (PR: 0.21; 95% CI: 0.03–0.85; *p* = 0.04), and being 17 years old (PR: 4.44; 95% CI: 1.48–13.39; *p* = 0.01) were associated with adolescent pregnancy. In the adjusted model, adolescents aged 15 years (aPR: 3.15; 95% CI: 1.04–9.52; *p* = 0.04) maintained a higher prevalence compared with those aged 14 years. Conclusions: An association was found between structural and intermediate social determinants of health and adolescent pregnancy in rural settings. These findings suggest the need for additional studies to better understand interventions targeting these associated factors.

## 1. Introduction

At the global level, sub-Saharan Africa and Latin America are the regions with the highest rates of adolescent pregnancy, showing higher figures among groups from rural areas [1,2]. Currently, maternal and neonatal mortality in Latin America constitutes a preventable challenge for health systems [3]. In Peru, the prevalence of adolescent pregnancy is intermediate compared with that of other Latin American countries, such as Venezuela and Ecuador [4,5]. However, a distinct situation exists in rural areas of the Peruvian Amazon, where the prevalence reaches 18.3%, compared with 6.1% in urban areas [5,6]. Culturally, these figures are often attributed to machismo, cultural opposition to contraceptive use, and early unions [7,8]. Likewise, limited access to education or school dropout among these populations [9] has repercussions on economic factors such as poverty and extreme poverty [10]. Peru shares with other Latin American countries the same cultural and socioeconomic factors that increase the likelihood of adolescent pregnancy [8,11]. However, the major difference is that these same factors largely overlap and tend to be more pronounced in rural areas with weak healthcare services [10]. Adolescent pregnancy represents a public health risk, primarily because of the increased incidence of maternal and neonatal complications [12,13], resulting from the immaturity of the vascular and uterine systems [12], which facilitates the development of preeclampsia, eclampsia, preterm birth, and low-birth-weight neonates [14].

Social determinants are the conditions in which people are born, grow, work, and age [15,16]. Therefore, they are widely recognized as responsible for health inequities, both within and between countries [17]. There are various ways to classify these determinants, primarily organizing them into structural determinants [18,19], which include the social, economic, and political factors that generate inequality and social stratification [19,20], and intermediate determinants, which encompass everyday living conditions and immediate behavioral lifestyle factors that directly impact health [20]. These determinants vary according to age group and the type of issue being addressed, and their understanding is essential for addressing complex problems such as adolescent pregnancy, where the interaction of multiple determinants directly influences its occurrence.

Previous studies have reported that the social determinants of health play a fundamental role in adolescent pregnancy. In Brazil, Colombia, and Peru, research has shown that structural determinants, such as low educational attainment and low wealth index, are associated with higher adolescent fertility rates [10,21,22]. Likewise, in Ecuador and Peru, intermediate determinants, such as low contraceptive use and limited knowledge of sexual health, have been reported to be associated with higher rates of adolescent pregnancy [8,23]. However, although studies have examined the health determinants associated with adolescent pregnancy, most are national, ecological, or qualitative studies, with no clear evidence of how these health determinants interact collectively. In particular, the Peruvian literature presents divergent findings, while one group of researchers prioritizes structural determinants such as poverty and illiteracy [10], another group emphasizes intermediate determinants such as contraceptive use and knowledge [8]. Therefore, the primary objective of this study was to analyze the association of structural and intermediate social determinants of health with adolescent pregnancy in rural settings of the Peruvian Amazon.

To address this objective, the following research questions are proposed: (1) Which structural social determinants of health are associated with adolescent pregnancy in rural communities of the Peruvian Amazon? (2) Which intermediate social determinants of health are associated with adolescent pregnancy in rural communities of the Peruvian Amazon?

## 2. Materials and Methods

An analytical cross-sectional study with a non-experimental design was conducted from June to December 2025 in the district of Lonya Grande, the Amazonas region. Lonya Grande is a predominantly rural district whose main socioeconomic activity is the production and commercialization of coffee; it has eight secondary educational institutions and ten basic rural health centers.

A consecutive non-probability sampling method was used. The recruitment sites for all adolescents aged 12 to 17 years were identified through the official open-access platforms, ESCALE (Ministry of Education, Lima, Peru) [24] and REUNIS (Ministry of Health, Lima, Peru) [25], both official open-access platforms of the Peruvian government. Data collection was carried out in the eight educational institutions and in the ten basic rural health centers of the district between June and December 2025. Participation invitations were extended consecutively to all adolescents who met the inclusion criteria and were present during the recruitment period at the data collection sites.

The sample size was determined using G*Power version 3.1.9.4 (Heinrich Heine University Düsseldorf, Germany), employing the Poisson regression module with the following parameters: α error prob = 0.05, statistical power = 0.90, and an effect size of f^2^ = 0.15 (equivalent to a prevalence ratio of 2.5, based on evidence documenting associations ranging from 1.5 to 2.5 between rural poverty and adolescent pregnancy) [26,27]. The sample size calculation yielded *n* = 53 participants. A total of *n* = 82 adolescents were recruited (128% of the planned sample size). Of these, 10 cases were excluded due to missing key data, resulting in a final analytical sample of *n* = 72 adolescents (30 pregnant and 42 non-pregnant).

To safeguard the well-being of the participants and uphold the ethical principles of autonomy, beneficence, and non-maleficence, the research was conducted in accordance with the principles of the Declaration of Helsinki. The study was reviewed and approved by the Institutional Research Ethics Committee (CIEI) of the Universidad Nacional Toribio Rodríguez de Mendoza through Resolution No. 00167, dated 5 June 2025. Prior to data collection, written informed consent was obtained from parents, mothers, or legal guardians, as well as assent from the adolescents, guaranteeing in both cases the right to withdraw from the study at any time without justification or retaliation.

During the study, the following inclusion criteria were established: (a) female adolescents aged 12 to 17 years residing in the district of Lonya Grande during 2025; (b) written informed consent from a parent or legal guardian and informed assent from the adolescent; and (c) willingness to participate and complete the questionnaire during the study period. Exclusion criteria were: (a) a diagnosis of neurological disability or cognitive impairment that, according to the National Registry of Persons with Disabilities (CONADIS, Ministry of Women and Vulnerable Populations, Lima, Peru), limited comprehension of the questionnaire; and (b) failure to complete the questionnaire in its entirety.

The questionnaire was administered digitally using mobile phones, tablets, and computers during scheduled sessions at the educational institutions and health centers in the district. For adolescents who reported having no personal devices, the research team provided equipment with access to the digital platform at these locations. Trained researchers were available during the administration to provide technical assistance and clarify any questions. The average duration of the application was approximately 45 min.

### 2.1. Adolescent Pregnancies

Pregnancy status was obtained through the following stages: (1) Self-report in questionnaire: Each adolescent was asked, Are you currently pregnant? Closed response options were given (yes/no/do not know). (2) Subsequent documentary verification: For adolescents who reported pregnancy (*n* = 30), verification was requested through available clinical documentation. During the administration of the questionnaire, participants’ Ministry of Health prenatal care card was requested, obtaining confirmation in *n* = 24 of the reported cases. Subsequently, for the remaining 6 adolescents who reported pregnancy but did not have the card available at the time, verification was carried out through the review of the medical record at the health facility, confirming pregnancy in *n* = 6 cases, resulting in complete verification of all *n* = 30 reported cases (100%).

No cases were identified of adolescents who reported pregnancy without accessible verifiable documentation. Nor were cases identified with negative self-report that presented clinical indicators of pregnancy. The collection of information from clinical documents was carried out following prior coordination and authorization from health personnel, respecting the confidentiality of the data in accordance with current regulations.

### 2.2. Social Determinants of Health

The questionnaire by Acosta Gil et al. (2022) [28] on social determinants was used, structured into two sections. The first section corresponds to structural determinants and includes five questions: type of health insurance, socioeconomic status, family type, father’s educational level, and mother’s educational level. The second section contains twelve questions on intermediate determinants, including age, religious practice, drug use, alcohol consumption, tobacco use, knowledge and use of contraceptives, family history of adolescent pregnancy, violence, supervision, communication, and family instability. The instrument presented categorized questions with closed-ended responses.

Reliability was verified through Cronbach’s alpha by the creators of the instrument, obtaining a result of α = 0.95, which demonstrated high reliability. Likewise, to demonstrate validity, the questionnaire was submitted to receive expert judgment by the Public Health Department of the Universidad del Norte, who determined its high validity [28].

### 2.3. Data Analysis

Data processing included descriptive and inferential statistics. Absolute frequencies and percentages were calculated for categorical variables. For the association analysis, the prevalence ratio (PR), appropriate for cross-sectional studies, was used. Crude prevalence ratios (PRs) were estimated using Poisson regression with robust variance, comparing each exposure category among adolescents with and without pregnancy. Ninety-five percent confidence intervals were obtained for each PR. Separate adjusted Poisson regression models with robust variance were constructed for the structural and intermediate social determinants of health, with all covariates included to estimate adjusted prevalence ratios (aPRs). Collinearity among the predictors was assessed using the Generalized Variance Inflation Factor (GVIF). All GVIF values were below 1.43 (structural determinants model: maximum GVIF = 1.14; intermediate determinants model: maximum GVIF = 1.43), which is well below the threshold of 5, indicating the absence of multicollinearity. The normality of continuous variables was assessed using the Shapiro–Wilk test; according to the results, Student’s t-test was applied for variables with a normal distribution, whereas the Mann–Whitney U test was used for those that did not meet this assumption. For categories with zero-frequency cells, the PR was estimated by applying the Haldane–Anscombe correction, and the *p*-value was obtained using Fisher’s exact test. All analyses were performed with 95% confidence intervals (95% CI) and a significance level of *p* < 0.05. Analyses were conducted using R version 2026.01.1+403 (Posit Software, PBC, Boston, MA, USA) [29], and the results were presented in tables prepared in Microsoft Excel 2019 version 2508 (Microsoft Corporation, Redmond, WA, USA) [30].

## 3. Results

The study population consisted of 30 pregnant adolescents and 42 non-pregnant adolescents. Regarding age, pregnant adolescents had a mean age of 15.07 ± 1.07 years, whereas non-pregnant adolescents had a mean age of 15.83 ± 1.02 years. Concerning socioeconomic status, 23 pregnant adolescents (76.67%) lived in conditions of extreme poverty, compared to 18 non-pregnant adolescents (42.86%). Regarding health coverage, the majority of pregnant adolescents (*n* = 26; 86.67%) had public health insurance, while only 23 non-pregnant adolescents (54.76%) had this type of coverage. Finally, in terms of family type, 16 pregnant adolescents (53.33%) came from nuclear families, compared to 28 non-pregnant adolescents (66.67%) with the same family structure, while 12 pregnant adolescents (40.00%) and 11 non-pregnant adolescents (26.19%) came from extended families (Table 1).

Among the structural determinants, extreme poverty was significantly more prevalent in pregnant adolescents (*n* = 23; 76.67%) compared to non-pregnant adolescents (n = 18; 42.86%), with adolescents in poverty having 2.48 times the prevalence of pregnancy (95% CI: 1.23–5.03; *p* = 0.004). Regarding type of health insurance, adolescents with public coverage had 3.05 times the prevalence of pregnancy compared to those with private insurance (95% CI: 1.21–7.72; *p* = 0.005), with 26 (86.67%) pregnant adolescents and 23 (54.76%) non-pregnant adolescents having public coverage. Regarding family type, no significant association with adolescent pregnancy was observed (*p* = 0.47), with most pregnant adolescents coming from nuclear families (*n* = 16; 53.33%), similar to non-pregnant adolescents (*n* = 28; 66.67%). Regarding paternal educational level, adolescents whose fathers had completed primary education had 4.65 times the prevalence of pregnancy (95% CI: 1.56–13.83; *p* = 0.01), with 20 (66.67%) pregnant adolescents in this category compared to 13 (30.95%) non-pregnant adolescents. Maternal educational level showed multiple associations: adolescents whose mothers had completed primary education had 2.47 times the prevalence of pregnancy (95% CI: 1.39–4.39; *p* = 0.002), while those whose mothers had no education had 2.78 times the prevalence (95% CI: 1.58–4.89; *p* = 0.0004) (Table 2).

Among the intermediate determinants with statistical significance, age was significantly associated with adolescent pregnancy (*p* = 0.02). Specifically, 17-year-old adolescents had the highest prevalence (*n* = 10; 33.33% of pregnant adolescents and *n* = 5; 11.90% of non-pregnant adolescents), corresponding to 4.44 times the prevalence of pregnancy (95% CI: 1.48–13.39; *p* = 0.01). Fifteen-year-old adolescents showed borderline significance (PR: 3.16; 95% CI: 1.00–9.93; *p* = 0.05), while 16-year-old adolescents did not show a significant association (PR: 2.96; 95% CI: 0.93–9.49; *p* = 0.07). Regarding religious practice, Catholic affiliation was a protective factor, with 19 (63.33%) pregnant adolescents identified as Catholic compared to 35 (83.33%) non-pregnant adolescents, who were associated with a lower prevalence of pregnancy (PR: 0.45; 95% CI: 0.27–0.75; *p* = 0.03). Alcohol consumption showed the strongest association with pregnancy: 10 (33.33%) pregnant adolescents reported alcohol consumption compared to only 1 (2.38%) non-pregnant adolescent, corresponding to 2.77 times the prevalence of pregnancy (95% CI: 1.85–4.16; *p* < 0.001). Contraceptive knowledge was significantly protective: only 1 (3.33%) pregnant adolescent reported knowledge of contraceptives compared to 33 (78.57%) non-pregnant adolescents, with contraceptive knowledge being associated with a lower prevalence of pregnancy (PR: 0.21; 95% CI: 0.03–0.85; *p* = 0.04) (Table 3).

Among the intermediate determinants without statistical significance, it was observed that, regarding drug use, all adolescents in both groups (*n*: 30 and *n*: 42; 100%) reported no use, and therefore it was not statistically evaluable. With respect to tobacco use, only one pregnant adolescent reported tobacco use (3.33%), while none of the non-pregnant adolescents reported such use, with no significant association identified (PR: 1.83; 95% CI: 0.78–4.27; *p* = 0.18). Regarding contraceptive use, 08 (26.67%) adolescents reported using contraceptives, compared to 08 (19.05%) among non-pregnant adolescents, showing no statistically significant difference (PR: 1.27; 95% CI: 0.71–2.29; *p* = 0.46). In terms of family history of adolescent pregnancy, 17 pregnant adolescents (56.67%) had such a history compared to 20 non-pregnant adolescents (47.62%), with no significant association identified (PR: 0.95; 95% CI: 0.82–1.09; *p* = 0.45) (Table 3).

Regarding family violence, only 01 (3.33%) pregnant adolescent reported experiencing violence, compared to 05 (11.90%) among non-pregnant adolescents, with no significant association identified (PR: 0.38; 95% CI: 0.06–2.32; *p* = 0.30). In relation to family supervision, 11 pregnant adolescents (36.67%) reported having supervision compared to 24 non-pregnant adolescents (57.14%), although this difference was not statistically significant (PR: 0.61; 95% CI: 0.34–1.09; *p* = 0.08). Regarding family communication, 16 pregnant adolescents (53.33%) reported regular communication, similar to non-pregnant adolescents (*n*: 21; 50.00%), with no significant association identified in the overall dimension (*p* = 0.60). Finally, regarding family instability, although 17 pregnant adolescents (56.67%) presented instability compared to 14 non-pregnant adolescents (33.33%), this association showed borderline statistical significance (PR: 1.72; 95% CI: 1.00–3.00; *p* = 0.05) (Table 3).

In the regression model adjusted for socioeconomic status, health insurance type, family structure, father’s educational level, and mother’s educational level, the variables that remained statistically significant were health insurance type and father’s educational level. Public health insurance was associated with an adjusted prevalence ratio (aPR) of 0.49 (95% CI: 0.36–0.66; *p* < 0.001), using private health insurance as the reference category. Adolescents whose fathers had completed only primary education had an adjusted prevalence ratio of 1.67 (95% CI: 1.15–2.42; *p* = 0.01) compared with the reference category. The remaining variables were no longer statistically significant after adjustment (Table 4).

In the adjusted model for the intermediate social determinants of health, none of the variables reached overall statistical significance. However, at the individual category level, adolescents aged 15 years (aPR = 3.15; 95% CI: 1.04–9.52; *p* = 0.04) and 17 years (aPR = 3.16; 95% CI: 1.03–9.71; *p* = 0.04) showed significantly higher prevalence ratios compared with those aged 14 years. The remaining variables—religious practice, alcohol consumption, contraceptive knowledge, family supervision, and family instability—did not reach statistical significance after adjustment (Table 5).

## 4. Discussion

In the bivariate model, structural determinants including health insurance type, socioeconomic status, and parental educational level were found to be associated with adolescent pregnancy, whereas among the intermediate determinants, the variables most strongly associated with adolescent pregnancy were alcohol consumption, contraceptive knowledge, and age. In the adjusted model, the variables that remained statistically significant among the structural determinants were health insurance type and the father’s educational level; however, the direction of the effect of health insurance type was reversed after adjustment. Regarding the intermediate determinants, no variable reached overall statistical significance. However, at the level of individual categories, adolescents aged 15 and 17 years had significantly higher prevalence ratios than those aged 14 years.

Health insurance type emerged as the predominant factor, with the prevalence of adolescent pregnancy being 3.05 times higher among adolescents with public health insurance than among those with private health insurance in rural settings. However, after adjustment for the remaining structural variables, the association was reversed, with an adjusted prevalence ratio of 0.49. This reversal of the findings represents a clear statistical suppression effect, also known as Simpson’s paradox. Similarly, socioeconomic status showed a significant association, with the prevalence of adolescent pregnancy being 2.48 times higher among adolescents living in extreme poverty than among those living in poverty; however, this association was no longer observed after adjustment. Previous studies have shown that adolescent pregnancy occurs more frequently among populations with low socioeconomic status or lower educational attainment [31,32]. Therefore, public health insurance or economic status initially acts as a marker of social vulnerability rather than as a causal factor [33], indicating that the association between health insurance type and adolescent pregnancy depends on the socioeconomic and family context in which adolescents live [32].

The scientific literature has shown that mothers with higher educational attainment are associated with better educational expectations and academic performance among their daughters [34], which is associated with a lower prevalence of early pregnancy [35,36]. Conversely, when parents have lower educational attainment, school dropout rates are higher [37] and are often associated with limited economic sustainability, which is in turn associated with early marriages or unions [38]. However, multiple studies have reported that parental educational attainment largely acts as a mediating variable rather than as a direct causal factor for adolescent pregnancy [39]. These findings are consistent with the present study, in which an association was identified in the bivariate analysis between both paternal and maternal educational attainment and a higher prevalence of adolescent pregnancy. However, after adjustment for other structural social determinants of health, only paternal educational attainment remained statistically significant, whereas maternal educational attainment was no longer associated with adolescent pregnancy. Furthermore, the findings regarding paternal educational attainment should be interpreted with caution because of the wide confidence intervals, which reflect uncertainty regarding its true effect on adolescent pregnancy. From this perspective, maternal educational attainment acts as a confounding variable associated with the coexistence of extreme poverty and public health insurance.

Regarding intermediate determinants, in the bivariate analysis, alcohol consumption emerged as a variable associated with a 2.77 times higher prevalence of adolescent pregnancy compared with adolescents who did not consume alcohol, whereas this association lost statistical significance in the model adjusted for other intermediate determinant variables. Several studies have highlighted that, during adolescence, the prefrontal cortex is still developing [40], so decision-making may be driven by impulses or pleasure [41,42]. When alcohol consumption is added to this process, the effects may be more pronounced and may be associated with greater acceptance of sexual relations with multiple partners [43,44]. However, alcohol consumption coexists with other family and sexual environment variables [45], so its role in adolescent pregnancy in rural communities represents a marker of vulnerability within these settings rather than an independent factor for adolescent pregnancy [46].

Another important intermediate determinant was age, with 17-year-old adolescents showing a 4.44 times higher prevalence of pregnancy compared with 14-year-old adolescents in the bivariate model. This association lost statistical significance in the adjusted model, reporting an adjusted prevalence ratio (aPR) of 3.16. Previous research indicates that older adolescents report an increased number of sexual encounters and a higher likelihood of marriage [47,48], representing multiple exposures that are associated with increased risk of at least one pregnancy [47]. However, other studies report that, with increasing age, adolescents experience greater freedom and socialization outside educational institutions [49,50], mainly due to family-related factors such as reduced parental supervision [50], making age a confounding variable for the prevalence of adolescent pregnancy [8,51]. In contrast, no statistical significance was observed for religion in relation to adolescent pregnancy in either the bivariate model or the model adjusted for other intermediate determinants in the present study. These results are consistent with previous research, where it has been reported that the influence of religion mainly depends on family environment dynamics in relation to these beliefs [52,53]. The findings suggest that the effect of religion may be related to other family environment variables rather than functioning as an independent determinant.

Contraceptive knowledge showed the strongest association in the bivariate model, with this association disappearing in the adjusted model, suggesting substantial confounding by other intermediate determinant variables. Previous studies have reported an increasing prioritization of sexual planning programs through health systems and support networks [54,55]. However, multiple vulnerable communities have cultural or religious contexts that limit the adoption of knowledge, associated with the emergence of myths such as infertility, which is associated with lower contraceptive uptake among the adolescent population in these communities [56]. This perspective made it possible to identify whether the variable of contraceptive knowledge level is susceptible to other population-specific variables; therefore, its use in public policies should be integrated with other intermediate determinants.

Similarly, while the level of knowledge about contraceptive methods showed statistical significance in the bivariate model, the variable of contraceptive use did not show significance in adolescent pregnancy in either the bivariate or the adjusted model. The discordance between contraceptive knowledge and use represents a critical gap between knowledge and action in rural settings. Several studies have reported that, despite adolescents being aware of contraceptive methods such as condoms or pills, they still have doubts regarding their effects, correct use, or effectiveness [57,58], associated with inconsistent or irregular use [59], where adolescents do not use contraceptives in all sexual encounters due to discrepancies or limited knowledge that undermine the ability to reach agreements within couples [60,61]. Likewise, in rural contexts, economic and geographic barriers limit the availability of contraceptives, or services are provided without privacy from parents or guardians, generating increased distrust among adolescents in these populations. Therefore, it is important to emphasize that the variables of contraceptive knowledge level and contraceptive use are strongly linked to other intermediate determinants, which influence the strength and direction of the association with adolescent pregnancy. Public interventions should comprehensively address the barriers specific to vulnerable communities, rather than addressing each determinant in a unified manner.

From this perspective, structural determinants such as health insurance type, socio-economic status, and parental educational level, along with intermediate factors such as alcohol consumption, age, and contraceptive knowledge, are associated with adolescent pregnancy. However, these variables should be considered in an integrated manner, as their association is often lost after adjustment for other family- and knowledge-related variables. This suggests that health interventions should be adapted to the specific characteristics and vulnerabilities of each population in order to effectively address adolescent pregnancy.

Despite these findings, several limitations should be considered. First, the relatively small sample size (n = 72) limits statistical power and reduces the predictive capacity of the estimates, particularly for variables such as paternal educational level and adolescent age, which show wide confidence intervals across several associations, reflecting that the true magnitude may vary considerably and restrict the generalizability of the findings to other vulnerable populations with cultural differences. Second, the cross-sectional design prevents the establishment of temporality, especially for variables such as contraceptive knowledge or use, in which potential reverse causality exists, whereby pregnancy itself may modify reported knowledge due to embarrassment, making it impossible to determine whether low contraceptive use led to pregnancy or whether pregnancy altered how contraceptive use is reported. Third, self-reported data collection is subject to information bias, particularly in rural contexts where information on alcohol consumption, sexual behavior, and contraceptive use is culturally sensitive. Finally, the lack of information on loss to follow-up or non-participating individuals prevents a full assessment of selection bias. Despite these limitations, the integrated approach to structural and intermediate determinants could improve the design of context-specific interventions for similar rural populations, where population size is relatively small. Therefore, future research is recommended to address these limitations through prospective longitudinal designs with larger sample sizes to strengthen causal inference and the generalizability of the results.

## 5. Conclusions

In conclusion, health determinants such as socioeconomic status and parental educational attainment, alcohol consumption, contraceptive knowledge, and adolescent age are generally associated with adolescent pregnancy in rural communities of the Peruvian Amazon. However, these factors are influenced by other variables that modify their statistical significance. Therefore, interventions targeting these populations should integrate multiple health factors into public policies, prioritize improved access to contraceptive methods, enhance adolescents’ privacy, and involve the family environment in sexual education. Despite the limitations inherent to the study’s cross-sectional design, the findings support the importance of conducting longitudinal studies with larger sample sizes to confirm the identified associations and strengthen the validity of the epidemiological conclusions.

## Figures and Tables

**Table 1:** Demographic data of pregnant and non-pregnant adolescents.

Variables	Pregnancy	
	Yes *n* = 30 (100%)	No *n* = 42 (100%)
Age, $\bar{x}$ ± SD	15.07 ± 1.07	15.83 ± 1.02
Socioeconomic Level, *n* (%)		
Poverty	07 (23.33)	24 (57.14)
Extreme poverty	23 (76.67)	18 (42.86)
Health Insurance Type, *n* (%)		
Private	4 (13.33)	19 (45.24)
Public	26 (86.67)	23 (54.76)
Family Type, *n* (%)		
Nuclear	16 (53.33)	28 (66.67)
Single parent	02 (6.67)	03 (7.14)
Extensa	12 (40.00)	11 (26.19)

Note: *n* = number of adolescents; % = percentage; $\bar{x}$ = mean; SD: standard deviation.

**Table 2:** Bivariate associations between structural social determinants of health related to adolescent pregnancy.

Variables	Pregnancy		PRs	95% CI	*p*
	Yes *n* = 30 (100%)	No *n* = 42 (100%)			
Socioeconomic Level, *n* (%)					0.004 *
Poverty (Ref.)	07 (23.33)	24 (57.14)	1.00	Ref.	Ref.
Extreme Poverty	23 (76.67)	18 (42.86)	2.48	1.23–5.03	0.004 *
Health Insurance Type, *n* (%)					0.005 *
Private (Ref.)	04 (13.33)	19 (45.24)	1.00	Ref.	Ref.
Public	26 (86.67)	23 (54.76)	3.05	1.21–7.72	0.005 *
Family Type, *n* (%)					0.47
Nuclear (Ref.)	16 (53.33)	28 (66.67)	1.00	Ref.	Ref.
Single parent	02 (6.67)	03 (7.14)	1.10	0.45–2.00	0.84
Extended	12 (40.00)	11 (26.19)	1.18	0.46–1.22	0.30
Educational Level of the Father, *n* (%)					0.01 *
High School Diploma (Ref.)	03 (10.00)	20 (47.62)	1.00	Ref.	Ref.
Completed Primary School	20 (66.67)	13 (30.95)	4.65	1.56–13.83	0.01 *
Incomplete Primary	02 (6.67)	03 (7.14)	3.07	0.68–13.82	0.15
Without education	05 (16.66)	06 (14.29)	3.48	1.01–12.02	0.05
Education Level of the Mother, *n* (%)					0.002 *
High School Diploma (Ref.)	14 (46.67)	36 (85.72)	1.00	Ref.	Ref.
Completed Primary School	09 (30.00)	04 (9.52)	2.47	1.39–4.39	0.002 *
Without education	07 (23.33)	02 (4.76)	2.78	1.58–4.89	0.0004 *

Note: *n* = number of adolescents; % = percentage; Ref = reference; PR: crude prevalence ratio; 95% CI = 95% confidence interval; * *p* < 0.05.

**Table 3:** Bivariate associations between intermediate social determinants of health related to adolescent pregnancy.

Variables	Pregnancy		PR	95% CI	*p*
	Yes *n* = 30 (100%)	No *n* = 42 (100%)			
Age years, *n* (%)					0.02 *
14 (Ref.)	03 (10.00)	17 (40.48)	1.00	Ref.	Ref.
15	09 (30.00)	10 (23.81)	3.16	1.00–9.93	0.05
16	08 (26.67)	10 (23.81)	2.96	0.93–9.49	0.07
17	10 (33.33)	05 (11.90)	4.44	1.48–13.39	0.01
Type of religious practice, *n* (%)					0.06
None (Ref.)	07 (23.33)	02 (4.76)	1.00	Ref.	Ref.
Catholic	19 (63.33)	35 (83.33)	0.45	0.27–0.75	0.03 *
Other Religions	04 (13.34)	05 (11.91)	0.57	0.25–1.28	0.34
Drug use, *n* (%)					NE
Yes	0 (0.00)	0 (0.00)	NE	NE	NE
No	30 (100.00)	42 (100.0)			
Alcohol consumption, *n* (%)					0.0004 *
No (Ref.)	20 (66.67)	41 (97.62)	1.00	Ref.	Ref.
Yes	10 (33.33)	01 (2.38)	2.77	1.85–4.16	<0.001 *
Tobacco consumption, *n* (%)					0.18
No (Ref.)	29 (96.67)	42 (100.0)	1.00	Ref.	Ref.
Yes	01 (3.33)	0 (0.00)	1.83 †	0.78–4.27 †	0.18
Use of contraceptives, *n* (%)					0.57
No (Ref.)	22 (73.33)	34 (80.95)	1.00	Ref.	Ref.
Yes	08 (26.67)	08 (19.05)	1.27	0.71–2.29	0.46
Contraceptive knowledge, *n* (%)					0.04 *
Unknown (Ref.)	29 (96.67)	09 (21.43)	1.00	Ref.	Ref.
Know	01 (3.33)	33 (78.57)	0.21	0.03–0.85	0.04 *
Family history of adolescent pregnancy, *n* (%)					0.77
No (Ref.)	13 (43.33)	22 (52.38)	1.00	Ref.	Ref.
Yes	17 (56.67)	20 (47.62)	0.95	0.82–1.09	0.45
Family violence, *n* (%)					0.39
Without violence (Ref.)	29 (96.67)	37 (88.10)	1.00	Ref.	Ref.
With violence	01 (3.33)	05 (11.90)	0.38	0.06–2.32	0.30
Family supervision, *n* (%)					0.10
Unsupervised (Ref.)	19 (63.33)	18 (42.86)	1.00	Ref.	Ref.
With supervision	11 (36.67)	24 (57.14)	0.61	0.34–1.09	0.08
Family communication, *n* (%)					0.60
Good (Ref.)	12 (40.00)	20 (47.62)	1.00	Ref.	Ref.
Regular	16 (53.33)	21 (50.00)	1.15	0.65–2.06	0.81
Bad	02 (6.67)	01 (2.38)	1.78	0.71–4.45	0.55
Family instability, *n* (%)					0.06
With stability (ref.)	13 (43.33)	28 (66.67)	1.00	Ref.	Ref.
Without stability	17 (56.67)	14 (33.33)	1.72	1.00–3.00	0.05

Note: *n* = number of adolescents; % = percentage; Ref: reference; PR = crude prevalence ratio; 95% CI = 95% confidence interval; * *p* < 0.05; NE = not evaluated; † Haldane–Anscombe correction applied due to presence of zero-value cell.

**Table 4:** Adjusted Poisson regression model with robust variance examining the association between structural social determinants of health and adolescent pregnancy.

Variables	Apr	95% CI	*p*
Socioeconomic Level			0.28
Poverty (Ref.)	1.00	-	-
Extreme Poverty	0.84	0.61–1.15	0.28
Health Insurance Type			<0.001 *
Private (Ref.)	1.00	-	-
Public	0.49	0.36–0.66	<0.001
Family Type			0.28
Nuclear (Ref.)	1.00	-	-
Single parent	0.68	0.17–2.74	0.20
Extended	0.84	0.57–1.24	0.37
Educational Level of the Father			0.04 *
High School Diploma (Ref.)	1.00	-	-
Completed Primary School	1.67	1.15–2.42	0.01 *
Incomplete Primary	2.18	0.85–5.56	0.11
Without Education	1.95	1.12–3.40	0.02 *
Education Level of the Mother			0.06
High School Diploma (Ref.)	1.00	-	-
Completed Primary School	0.48	0.21–1.09	0.08
Without Education	0.37	0.12–1.11	0.08

Note: Poisson regression model with robust variance. aPR: Prevalence ratio adjusted for the other variables included in the model (socioeconomic status, health insurance type, family structure, father’s educational level, and mother’s educational level). Collinearity among the predictors was assessed using the Generalized Variance Inflation Factor (GVIF). All GVIF values were below 1.14, indicating no evidence of collinearity among the variables included in the model. 95% CI: 95% confidence interval. *p*: Overall *p*-value obtained using the Wald test with robust variance. * = *p* < 0.05.

**Table 5:** Adjusted Poisson regression model with robust variance examining the association between intermediate social determinants of health and adolescent pregnancy.

Variables	aPR	95% CI	*p*
Age years			0.21
14 (Ref.)	1.00	-	-
15	3.15	1.04–9.52	0.04 *
16	3.09	0.96–9.97	0.06
17	3.16	1.03–9.71	0.04 *
Type of religious practice			0.26
None (Ref.)	1.00	-	-
Catholic	0.68	0.40–1.16	0.16
Other religions	0.97	0.41–2.31	0.95
Alcohol consumption			0.08
No (Ref.)	1.00	-	-
Yes	1.85	0.94–3.65	0.08
Contraceptive knowledge			0.34
Unknown (Ref.)	1.00	-	-
Know	2.49	0.38–16.12	0.34
Family supervision			0.10
Unsupervised (Ref.)	1.00	-	-
With supervision	1.58	0.92–2.72	0.10
Family instability			0.17
With stability (ref.)	1.00	-	-
Without stability	1.39	0.87–2.22	0.17

Note: Adjusted Poisson regression model with robust variance, adjusted for all variables included. aPR: adjusted prevalence ratio; 95% CI: 95% confidence interval; Ref.: reference category. Collinearity was assessed using the Generalized Variance Inflation Factor (GVIF); all values were below 1.43. The overall *p*-value was calculated using the Wald test with robust (sandwich) variance. The individual *p*-value corresponds to each category, relative to the reference. * = *p* < 0.05.

## Data Availability

The data will be available upon request from the corresponding author.

## Abbreviations

The following abbreviations are used in this manuscript:

PR	Prevalence Ratio
aPR	adjusted Prevalence Ratio
MCAR	Missing Completely at Random
Ref.	Reference
NE	Not Evaluated
